# Advancing research on *Blastocystis *through a One Health approach

**DOI:** 10.12688/openreseurope.18046.1

**Published:** 2024-07-12

**Authors:** Anastasios D. Tsaousis, Eleni Gentekaki, C. Rune Stensvold

**Affiliations:** 1 Laboratory of Molecular and Evolutionary Parasitology, School of Biosciences, University of Kent, Canterbury, CT2 7NJ, UK; 2 Department of Veterinary Medicine, University of Nicosia School of Veterinary Medicine, Nicosia, Nicosia, Cyprus; 3 Department of Bacteria, Parasites and Fungi, Artillerivej 5, Statens Serum Institut, Copenhagen, DK-2300, Denmark

**Keywords:** Blastocystis, One Health, Epidemiology, Diagnostics, Microbiome, 'Omics data, in vivo studies, Public Health

## Abstract

*Blastocystis* is the most prevalent intestinal eukaryotic microorganism with significant impacts on both human and animal health. Despite extensive research, its pathogenicity remains controversial. The COST Action CA21105, " *Blastocystis* under One Health" (OneHealthBlastocystis), aims to bridge gaps in our understanding by fostering a multidisciplinary network. This initiative focuses on developing standardised diagnostic methodologies, establishing a comprehensive subtype and microbiome databank, and promoting capacity building through education and collaboration. The Action is structured into five working groups, each targeting specific aspects of *Blastocystis* research, including epidemiology, diagnostics, 'omics technologies, *in vivo* and *in vitro* investigations, and data dissemination. By integrating advances across medical, veterinary, public, and environmental health, this initiative seeks to harmonise diagnostics, improve public health policies, and foster innovative research, ultimately enhancing our understanding of *Blastocystis* and its role in health and disease. This collaborative effort is expected to lead to significant advancements and practical applications, benefiting the scientific community and public health.

## Plain Language Summary

*Blastocystis* is a common microorganism found in the intestines of humans and animals. Its role in causing disease is still debated among scientists. The " *Blastocystis* under One Health" initiative aims to unite experts from human medicine, veterinary science, and environmental science to better understand this microorganism and its health effects.

The project focuses on improving diagnostic methods, creating a comprehensive database of *Blastocystis* samples, and analysing its genetic and molecular makeup. Researchers will also study how *Blastocystis* interacts with other gut microbes and impacts gut health. Additionally, the initiative aims to educate healthcare professionals and the public about *Blastocystis*.

By working together, scientists hope to develop better ways to diagnose, treat (if necessary), and/or prevent *Blastocystis* infections, ultimately protecting both human and animal health and enhancing our understanding of this widespread microorganism.

## Introduction

We are writing to emphasise the importance of comprehensive research on *Blastocystis* using a One Health approach. Integrating medical, veterinary, public, and environmental health research is essential to understanding the role of *Blastocystis* in health and disease. Despite its prevalence in over one billion people ^1^ and numerous animal species, the role of *Blastocystis* in health and disease remains controversial ^2^. Current studies offer conflicting views, necessitating a unified approach to unravelling its true impact on health ^3^.

## Background and importance

**Figure 1.**
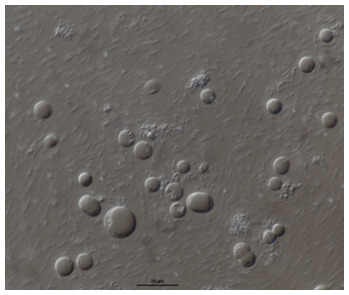
Overview of *Blastocystis* cells in xenic culture. Microscopy picture provided by Dr Vasana Jinatham (Mae Fah Lunag University, Thailand).

*Blastocystis* ( Figure 1) is a ubiquitous intestinal eukaryotic microbe found in both humans and animals. Emerging data suggest that its prevalence in herbivorous animals is higher than previously thought ^4, 5^. Despite numerous studies, there is still significant debate regarding its pathogenicity. While some researchers consider *Blastocystis* to be a harmless commensal organism, others consider it as a cause of gastrointestinal symptoms ^3, 6^. This ambiguity highlights the need for a comprehensive and integrated research approach under the One Health perspective ^7^.

The European Cooperation in Science and Technology (COST) Action CA21105, titled " *Blastocystis* under One Health" (OneHealthBlastocystis), seeks to address these challenges by fostering a multidisciplinary network of researchers and professionals ^8, 9^. This initiative aims to harmonise diagnostic methodologies, establish a comprehensive databank and biobank, and promote information sharing and capacity building among stakeholders. By integrating advances across medical, veterinary, public, and environmental health domains, we can develop a more nuanced understanding of *Blastocystis* and its implications for health and disease.

## Objectives and goals

The primary objective of the COST Action CA21105 ( 8 see MoU) is to create a multidisciplinary network to enhance research on *Blastocystis*. The specific goals of this initiative include:

- Developing standardised methodologies for the detection and subtyping of *Blastocystis* to ensure consistency and reliability in research findings.

- Establishing a comprehensive *Blastocystis* subtype databank and biobank and microbiome databank to consolidate existing data and facilitate new research.

- Promoting capacity building through training schools, workshops, and short-term scientific missions (STSM) to expand expertise and foster collaboration among researchers, clinicians, and public health professionals.

- Disseminating knowledge and fostering communication with veterinarians, physicians, and the general public to increase awareness and understanding of *Blastocystis*.

## Working groups and their aims

**Figure 2.**
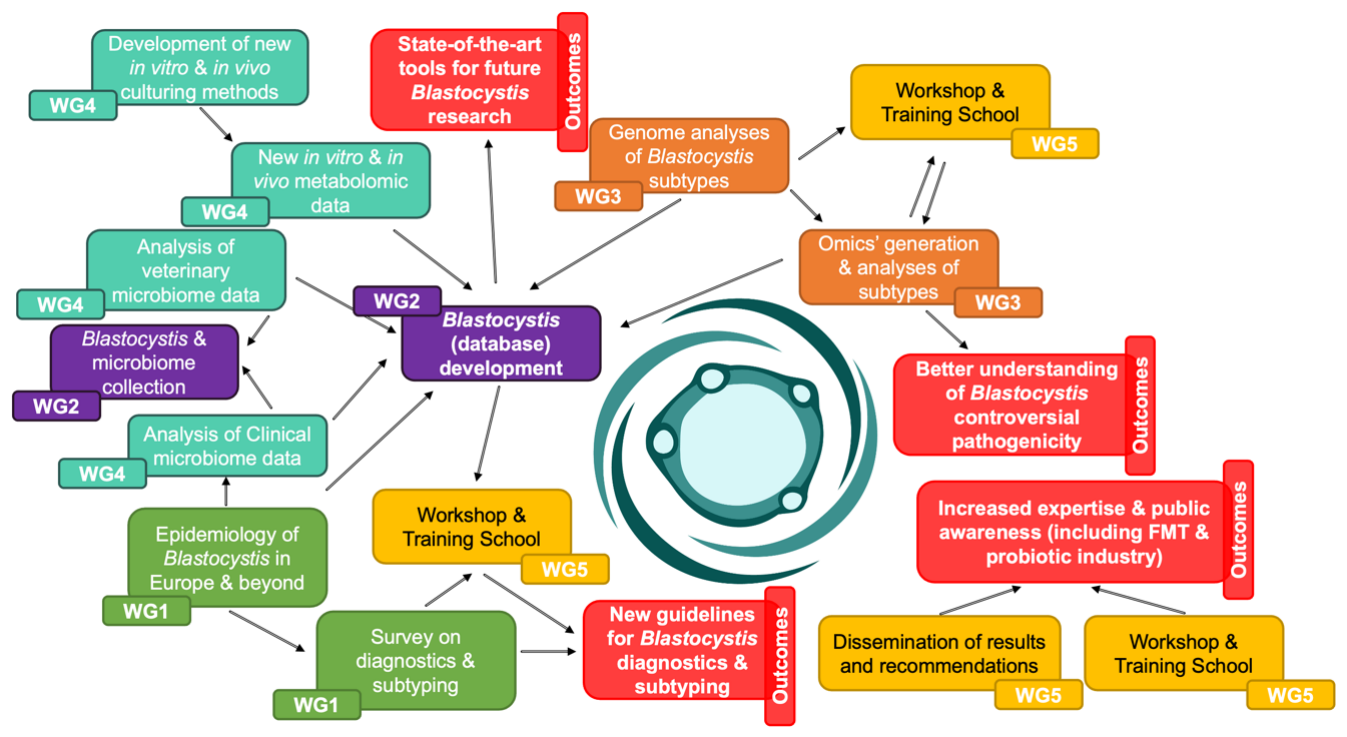
PERT chart summarising the holistic approach of the *Blastocystis* under One Health COST Action.

The initiative is structured into several working groups ( Figure 2), each with specific aims and tasks to address different aspects of *Blastocystis* research:

### WG1: Mapping *Blastocystis* epidemiology and diagnostics

- Objective: Harmonise diagnostic methodologies and interpretive criteria across Europe.

- Tasks: Review current practices, introduce quality assurance mechanisms, and assess microbiological practices. This group aims to create a common understanding of *Blastocystis* under the One Health umbrella by identifying and addressing gaps in current diagnostic approaches.

### WG2: *Blastocystis* collection and database

- Objective: Establish and maintain both a databank and biobank of *Blastocystis* subtypes and microbiome data.

- Tasks: Develop material and data transfer agreements, create a comprehensive database including epidemiological and ‘omics data along with a subtypes database. The databank, subtypes database, along with a subtype’s biobank, will be crucial resources for researchers, providing access to standardised data and facilitating collaborative studies.

### WG3: *Blastocystis* ‘omics generation and analyses

- Objective: Analyse genomic, proteomic, and metabolomic data to better understand *Blastocystis*.

- Tasks: Organise and analyse existing genomic data, generate new ‘omics data, and integrate findings into the database. This group will focus on understanding the molecular and genetic aspects of *Blastocystis*, contributing to a deeper knowledge of its biology and potential pathogenicity.

### WG4: *Blastocystis* in vivo and in vitro investigations

- Objective: Investigate the role of *Blastocystis* in the gut microbiome and develop culturing methods.

- Tasks: Analyse microbiome data from humans and animals, develop protocols for culturing *Blastocystis*, and compare metabolomic data. This group aims to explore the interactions between *Blastocystis* and other gut microbes, providing insights into its ecological role and potential health effects.

### WG5: Dissemination and education

- Objective: Promote knowledge dissemination and educational activities.

- Tasks: Develop an Action website, produce teaching materials, coordinate training schools and workshops, and publish an open-access book on *Blastocystis* research. This group will ensure that the findings and methodologies developed during the project are widely shared and accessible to both the scientific community and the public.

## Capacity building

The CA21105 COST Action aims to build a sustainable network of stakeholders from various disciplines and countries. Focus areas include:

- Facilitating training schools, workshops and STSMs to expand expertise. By organising training schools and workshops, the Action will provide opportunities for researchers, clinicians, and other stakeholders to enhance their skills and knowledge in *Blastocystis* research.

- Encouraging involvement of young researchers and innovators, especially from countries with limited resources. This initiative places a strong emphasis on supporting early-career researchers and ensuring that expertise is built across a wide geographical area, including regions with fewer resources.

## Future prospects

The Action outlines future research initiatives to understand *Blastocystis* better and support the development of potential market applications (e.g. inclusion of *Blastocystis* in faecal microbiota transplantations or as probiotic formulations in both humans and other animals). The comprehensive databank and standardised methodologies developed by this initiative will serve as valuable resources for future studies. By harmonising diagnostics and fostering collaboration, the Action aims to improve public health policies and advance our understanding of *Blastocystis*.

Specific future prospects include:

- Developing novel hypotheses to test the role of *Blastocystis* in the gut ecosystem, health, and disease. This could lead to ground-breaking discoveries about its interactions with other microorganisms and its impact on gut health.

- Expanding knowledge on the prevalence and subtype distribution of *Blastocystis* in humans and animals. This information is crucial for understanding transmission dynamics and identifying potential zoonotic and environmental sources of infection.

- Generating new insights into the biology and ecology of *Blastocystis*. Researchers can gain a deeper understanding of its role in health and disease by studying its interactions with the gut microbiome and its responses to different environmental conditions.

- Developing new diagnostic tools and treatment guidelines. The standardised methodologies and comprehensive databank will enable the development of more accurate and efficient diagnostic tools and evidence-based treatment guidelines.

- Promoting the use of *Blastocystis* as a model organism and potential probiotic. The initiative will explore the potential of *Blastocystis* to be used as a model organism for studying gut microbiota and as a probiotic for promoting gut health.

## Conclusion

By adopting a One Health approach, this initiative will significantly advance our understanding of *Blastocystis*. We call on the global research community to collaborate in this multidisciplinary effort to improve health outcomes. The COST Action CA21105, " *Blastocystis* under One Health" (OneHealthBlastocystis), represents a unique opportunity to bridge gaps in current knowledge, harmonise diagnostic methodologies, and promote innovative research. Through the collaborative efforts of researchers, clinicians, public health professionals, and other stakeholders, we can develop a more comprehensive understanding of *Blastocystis* and its role in health and disease. This initiative will not only contribute to scientific knowledge and development but also have practical applications in public health, diagnostics, and treatment.

We urge readers to support this crucial initiative and encourage researchers from diverse fields to join this collaborative effort. Together, we can achieve significant advancements in *Blastocystis* research and improve health outcomes for people and animals worldwide.

## Disclaimer

The views expressed in this article are those of the authors. Publication in *Open Research Europe* does not imply endorsement by COST Actions.

## Ethics and consent

Ethical approval and written consent were not required.

## Data Availability

No data are associated with this article.
