# Supplementary material for: Advancing research on Blastocystis through a One Health approach
Source: Open Res Eur. 2024 Jul 12;4:145. [Version 1] doi: 10.12688/openreseurope.18046.1 (PMC11294803; doi:10.12688/openreseurope.18046.1)
Supplement: Peer review history

# Reviewer report: Reviewer response for version 1

**DOI:** 10.21956/openreseurope.19502.r42139

**Published:** 2024-08-05

**Authors:** Arutchelvan Rajamanikam

**Affiliations:** 1 University Malaya, Kuala Lumpur, Malaysia

The open letter clearly describes and explains the purpose of studying Blastocystis in One Health context while also addressing the different aspects of Blastocystis.

 It would be a good idea to include the current awareness & understanding of Blastocystis in participating countries as this will clearly show the gaps in research that this action purports to fulfil. 

 The authors may also consider expanding a bit more on the purposes and importance of each working group in the field of Blastocystis research. 

 I hope the authors would give equal importance on the influence of microbial flora on Blastocystis. It should not left out in understanding of Blastocystis-microbiota interaction. 

 The article is otherwise scientifically sound and concise.

 P/S: there is a spelling mistake in figure 1, name of the university is spelled wrongly.

Where applicable, are recommendations and next steps explained clearly for others to follow? (Please consider whether others in the research community would be able to implement guidelines or recommendations and/or constructively engage in the debate)

Yes

Does the article adequately reference differing views and opinions?

Yes

Are all factual statements correct, and are statements and arguments made adequately supported by citations?

Yes

Is the rationale for the Open Letter provided in sufficient detail? (Please consider whether existing challenges in the field are outlined clearly and whether the purpose of the letter is explained)

Yes

Is the Open Letter written in accessible language? (Please consider whether all subject-specific terms, concepts and abbreviations are explained)

Yes

Reviewer Expertise:

Intestinal parasitology, Gut microbiome, host-parasite interaction

I confirm that I have read this submission and believe that I have an appropriate level of expertise to confirm that it is of an acceptable scientific standard.

# Reviewer report: Reviewer response for version 1

**DOI:** 10.21956/openreseurope.19502.r42140

**Published:** 2024-08-01

**Authors:** Małgorzata Lepczyńska

**Affiliations:** 1 Department of Medical Biology, School of Public Health, Collegium Medicum, University of Warmia and Mazury, Żołnierska 14 C, Poland

The Letter is generally well-organized, very clearly and logically written. Overall, the initiative is undoubtedly impactful and crucial for World’s healthcare landscape.

 The initiative addresses a critical gaps in the current problems in methodology of *Blastocystis* detection as well as its biology, pathogenicity and treatment which may lead to misunderstanding of its true nature.

 This Open Letter introduces to the reader the main challenges we are facing in research on *Blastocystis*. The authors pointed out some important concepts as:

 - developing methodologies for the detection and subtyping of *Blastocystis* so the research findings can be more reliable than the results indexed previously;

 - establishing a *Blastocystis* subtype and microbiome databank to make a new research easier to all scientists as well as to consolidate existing data;

 - promoting training schools and workshops to expand expertise among researchers and health professionals;

 - disseminating knowledge and improving communication between researchers and veterinarians, physicians, and the general public to increase awareness and understanding of *Blastocystis* to finally not underestimate its power.

 The structure of several working groups is presented on the figure, what is a helpful addition that makes the content more accessible and clear.

 The authors underlined how important the multidisciplinary program is, according to the nature of mysterious *Blastocystis*. Because the pathogenicity or commensal nature depends on the *Blastocystis* subtype, the geographic area or the gut microbiota composition, it is necessary to invite a large group of researchers and professionals of a various disciplines.

 In the Letter the authors make the reader aware of the initiative importance: many medical branches will develop – starting from laboratory staff (methodology) through the researchers, public health professionals, ending on the clinicians and veterinarians according to the treatment.

 The authors invite the reader to support their initiative as well as the researchers to join as a part of that cooperative community so the *Blastocystis* research network may be larger and give more effective results.

 I strongly recommend approval of this manuscript.

Where applicable, are recommendations and next steps explained clearly for others to follow? (Please consider whether others in the research community would be able to implement guidelines or recommendations and/or constructively engage in the debate)

Not applicable

Does the article adequately reference differing views and opinions?

Yes

Are all factual statements correct, and are statements and arguments made adequately supported by citations?

Yes

Is the rationale for the Open Letter provided in sufficient detail? (Please consider whether existing challenges in the field are outlined clearly and whether the purpose of the letter is explained)

Yes

Is the Open Letter written in accessible language? (Please consider whether all subject-specific terms, concepts and abbreviations are explained)

Yes

Reviewer Expertise:

medical parasitology (mainly Blastocystis), microbiology, medical biology, medical education

I confirm that I have read this submission and believe that I have an appropriate level of expertise to confirm that it is of an acceptable scientific standard.

# Response

**Published:** 2024-08-01

**Authors:** Anastasios Tsaousis

**Affiliations:** School of Biosciences, University of Kent, Canterbury, England, UK

We would like to thanks the reviewer for the positive response on our manuscript.  Kind regards, Anastasios Tsaousis

# Reviewer report: Reviewer response for version 1

**DOI:** 10.21956/openreseurope.19502.r42144

**Published:** 2024-07-30

**Authors:** Anna Lass

**Affiliations:** 1 Medical University of Gdańsk, Gdynia, Poland

In my opinion, the article can be indexed as it is. It does not require linguistic or substantive corrections.

 The letter presents the assumptions of the COST Action CA21105 " *Blastocystis *under One Health" in an accessible and concise way, emphasizing the importance of the topic undertaken jointly within an international, interdisciplinary network. The ideas, goals and challenges of the individual working groups established in this project are clearly presented as well.

 The authors of the letter rightly highlight the crucial importance of integrating medical, veterinary, public and community research in understanding the nature of *Blastocystis*, thereby clearly demonstrating the appropriateness of undertaking multicenter cooperation in the spirit of the "One Health" approach.

Where applicable, are recommendations and next steps explained clearly for others to follow? (Please consider whether others in the research community would be able to implement guidelines or recommendations and/or constructively engage in the debate)

Yes

Does the article adequately reference differing views and opinions?

Yes

Are all factual statements correct, and are statements and arguments made adequately supported by citations?

Yes

Is the rationale for the Open Letter provided in sufficient detail? (Please consider whether existing challenges in the field are outlined clearly and whether the purpose of the letter is explained)

Yes

Is the Open Letter written in accessible language? (Please consider whether all subject-specific terms, concepts and abbreviations are explained)

Yes

Reviewer Expertise:

parasitology; epidemiological studies on the occurrence of Blastocystis in human, animal and environmental material, with particular emphasis on molecular and phologenetic studies

I confirm that I have read this submission and believe that I have an appropriate level of expertise to confirm that it is of an acceptable scientific standard.
